# Black Kites on a flyway between Western Siberia and the Indian Subcontinent

**DOI:** 10.1038/s41598-022-09246-1

**Published:** 2022-04-02

**Authors:** Ivan Literák, Jan Škrábal, Igor V. Karyakin, Natalya G. Andreyenkova, Sergey V. Vazhov

**Affiliations:** 1 Department of Biology and Wildlife Diseases, Faculty of Veterinary Hygiene and Ecology, University of Veterinary Sciences Brno, Palackého tř. 1946/1, 61242 Brno, Czech Republic; 2 Sibecocenter, LLC, Pirogova Str. 20/2, 630090 Novosibirsk, Russia; 3 Institute of Molecular and Cellular Biology SB RAS, Acad. Lavrentiev Ave. 8/2, 630090 Novosibirsk, Russia; 4 Shukshin Altai State University for Humanities and Pedagogy, Korolenko Str. 53, 659333 Biysk, Russia

**Keywords:** Ecology, Zoology

## Abstract

The Black Kite (*Milvus migrans*) is one of the most widespread raptors in the world. The Palaearctic is populated by two migrating subspecies, *Milvus migrans migrans* and *Milvus migrans lineatus*, in the western and eastern part of this realm, respectively. There is a large intergradation zone of *M. m. migrans/M. m. lineatus* in-between. Although the migration routes of *M. m. migrans* from Europe to Sub-Saharan Africa and the Middle East are well known, detailed information about migration routes of Black Kites from intergradation zone are missing. Using satellite telemetry we are able to fill this gap in our knowledge of these birds. We tagged with GPS/SMS/GPRS telemetry loggers 13 and 6 Black Kite *pulli* in lowland around Biysk (Altai Krai, Russia) and in mountains around Kosh-Agach (Altai Republic, Russia), respectively*.* After fledging, Black Kites from both subpopulations stayed in a small, non-overlapping areas and then migrated to southern Asia through narrow corridors. Black Kites originating from Biysk migrated through the Western Circum-Himalayan Corridor. Black Kites originating from Kosh-Agach used the Trans-Himalayan Corridor crossing the Himalayas in altitudes of up to 6256 m asl. The average total distance travelled of Black Kites from both subpopulations was 9166 km without any significant differences between these subpopulations. Timing of both spring and autumn migration did not vary along different age groups. Black Kites from both subpopulations wintered in low elevations of Pakistan and India. Birds wintered on average for 190 days, and the mean area of individual home ranges in winter was 4704 km^2^. During the breeding period, birds dwelled in south-western Siberia, where they spent on average 125 days with an average home range size 3537 km^2^. We found that ontogenetic shifts in migratory behaviour of Black Kites from Eastern Russia differ from those in population/subspecies in Europe. Black Kites crossing the Himalayas fly and, moreover, stay for hours resting at night in the environment of mountains at altitudes over 5000 m.

## Introduction

The Black Kite (*Milvus migrans*) is one of the most widespread raptors occurring in Eurasia, Africa, and Australia^1,2^. It shows unique ecological flexibility and can inhabit many habitats, including human-affected landscapes, using variable food sources as an opportunistic predator and scavenger^3–6^. The Palaearctic is populated by two subspecies, *Milvus migrans migrans* and *Milvus migrans lineatus* in the western and eastern part of this realm, respectively^7–10^. Both subspecies carry specific morphological features that enables their recognition. The ranges of the Palearctic subspecies contact each other with an opportunity for mutual hybridizations. The intergradation zone of *M. m. migrans/M. m. lineatus* covers large areas of Eastern Europe, Kazakhstan, and West Siberia and is gradually expanding^1,11–15^. The most active is the westward expansion so that the birds with the characters of *M. m. lineatus* already occur in Iberian Peninsula^16^. Black Kites of these two subspecies seem to cross freely since birds in the intergradation zone exhibit a whole range of intermediate characters^13^.

The Palaearctic *M. m. migrans* and *M. m. lineatus* are seasonal migrants. *M. m. migrans* from Europe winter in sub-Saharan Africa and the Middle East. Its migration routes are well known and its circannual variations in the movement patterns have been extensively reviewed^6^. During migration, tens of thousands of *M. m. migrans* are observed migrating across the Straits of Gibraltar, along the eastern coast of the Black Sea and in the Middle East; while, substantial numbers cross the central Mediterranean (including Italy, Sicily, and Tunisia) and the Bosporus^6,17^. Few birds cross the Mediterranean Sea between the Peloponnese, Crete, and Libya^6^. The crossing of large water bodies of the Mediterranean Sea and the Black Sea seems to be challenging environmental obstacles for *M. m. migrans* on the flyway from Europe to Africa^6,18,19^. In contrast to European Black Kites, migration routes of Black Kites from the Siberian part of Russia are mainly unknown. Some observations of Black Kites in autumn around Novosibirsk and the Kuznetsky Alatau Mountains indicated their direction of autumn migration to Kazakhstan, and aggregations of Black Kites on their autumn migration route through Kazakhstan were reported from Chokpak Pass^20–22^.

The migration of *M. m. lineatus* further east is also poorly studied. Based on the phenotypes of observed birds, *M. m*. *lineatus* overwinters in India, Indo-China, and China^23^. Black Kites (pure *M. m. lineatus*?) from the eastern part of Mongolia overwinter in north-eastern India and Myanmar^24,25^. Black Kites, supposedly originating from the intergradation zone between *M. m. migrans* and *M. m. linetus* migrate traditionally to winter in Iraq, Iran, and western Pakistan^11^. Between 2014 and 2018, Black Kites called Black-eared Kites (English translation for *M. m. lineatus*) had been tagged with GPS transmitters at the landfill in Dehli, India, but unfortunately it was not specified if Kites originated from the intergradation zone between *M. m. migrans* and *M. m. lineatus* or pure *M. m. lineatus*^26^. These pre-adult and adult Kites migrated for 3300–4800 km along a narrow corridor between Dehli (winter quarters) and southern Siberia and western Mongolia (summer including supposedly breeding quarters). This study confirmed that kites can cross the Himalayas at elevations up to more than 6500 m asl by the K2 of the Karakoram Range and can travel long periods at elevations above 3500 m asl Previously, Black Kites were regularly observed during migration at various watch-sites in the Himalayan ridge in northwestern India, Nepal and the Tibetian Autonomous Region, western China^27^. These recent insights suggests that there may be a larger migration of Kites from the integration zone in Siberian Russia across the Himalayas and into the Indian Subcontinent than assumed so far.

Black Kites are known to cross different natural barriers and face many challenging conditions along their migration^18^. Among atmospheric conditions, wind is known to strongly influence the speed of migration specifically during long-distance migration and overcoming of natural barriers^28–30^. Good wind condition, mainly prevailing tailwind, may induce a decision to depart^31^. Many studies have shown that both flapping and soaring migrants travel significantly faster when flying with tailwinds and are slowdown by headwind and crosswinds^32,33^. Thanks to technological progress, we can use tracking technology to record the migratory routes of wild birds and other animals. What is more, we are able to determine from weather records the atmospheric conditions at the locations where the birds were recorded^34^. By this approach we can reconstruct how key factors such as wind affected birds during migration^30^, whereby we expect Kites to cross a harsh barrier such as the Himalayas mainly in supportive seasonal winds.

Raptors of several species were observed to migrate across the Himalayan region, and based on all data available, there were characterised four movement patterns of raptor migrating in this area: (1) Western Circum-Himalayan Corridor, (2) Eastern Circum-Himalayan Corridor, (3) East-to-West Southern Corridor and (4) Trans-Himalayan Corridor^27^. Bar-headed Geese (*Anser indicus*) are known to cross the high altitudes of Himalayas within one day, using mainly powered flapping flight even during steep descents^35^. Bar-headed Geese often migrate at night and in the early morning when the predominant winds travel downslope, therefore they cannot take an advantage of upslope wind during ascent and have to rely on their own power sources^35^. Their bodies show many specialized physiological adaptations, that help with harsh conditions (e.g. hypoxia) during the crossing. In the contrast to Bar-headed Geese we assume that Black Kite, as diurnal soaring raptor, might cross the Himalayas during day using upslope tailwinds to ascent and glide, increasing its airspeed, in order to use minimum energy and lower the oxygen requirement in hypoxic environment, because at slow airspeeds, a large amount of power is needed to support the bird's weight against gravity. Although Black Kite has a similar wingspan as Bar-Headed Geese, their body mass is 4–5 times smaller. Because of the differences in flight strategy, size and strength we expect that the favourable wind (prevailing tailwind), affecting bird’s speed, may play a major role in Black Kites’ timing and overall success of crossing over the Himalayas as they cannot persist in powered flapping flight as Bar-headed Geese.

Previous study regarding a natal dispersal of *M. m. migrans* in Europe used positions obtained on 31 January (winter ground) and 30 June (summer ground) to show how individual birds changed their migratory behaviour with age and experience during their early life years^18,19^. Surprisingly for birds in 2cy, it was found that summer quarters occurred at lower latitudes than predicted and a substantial proportion of birds of this age remained in their African winter quarters. Some birds in their 3cy returned to spend the summer period in the natal area, but many of these birds remained at lower latitudes north of their winter quarters. Accordingly, we expect similar behavioural changes to occur in juvenile Kites from Eastern Russia.

We studied Black Kites fledged in southwestern Siberia, Russia, which were tagged as *pulli* (a nestling that is not yet able to fly) with GPS transmitters on nests in (a) lowland area near Biysk, Altai Krai populated by Kites from the intergradation zone between *M. m. migrans* and *M. m. lineatus*, (b) in a mountainous area near Kosh-Agach, Altai Republic populated by supposedly pure *M. m. lineatus.* The aims of the study were (a) examine the genetic background of birds from these areas, (b) to reveal and to compare migration routes of Kites from these two populations, (c) to define the timing of autumn and spring migrations, (d) to characterise sizes of their post-fledging area, home ranges in wintering quarters and summering (breeding) ranges, and (e) to study, including weather conditions, the way how Kites crossed the extremely high elevations of the Himalayas as the leading environmental obstacle on their migration.

## Materials and methods

### Birds

In total, 19 Black Kites (11 females, 8 males) from breeding populations in Western Siberia, Russia, were investigated in this study. Kites originated from two spatially separated breeding subpopulations. One subpopulation represents birds hatched in lowland around Biysk (Altai Krai), the second subpopulation represents birds hatched in the mountains around Kosh-Agach (Altai Republic) near the Mongolian border (Table 1).

**Table 1:** Black Kites from western Siberia, Russia tracked with telemetry devices. All birds were tagged as *pulli*.

Black Kite ID	Logger No	Ring number	Date of tagging	Date of last observation	Sex	*cytB *haplo-group	*cytB *haplo-type	Nest location (coordinates)	Days of observation	Number of GPS fixes	Siblings
K1	KITE10	C 835581	06.07.2018	28.07.2018	F	B1	B19	52.53 N, 85.56 E	22	188	–
K2	KITE21	C 553828	07.07.2018	14.08.2018	M	B1	B14	52.50 N, 85.46 E	38	147	–
K3	KITE23	C 835582	07.07.2018	08.09.2020	M	B1	B6.1	52.55 N, 85.46 E	794	1656	–
K4	KITE24	C 835583	07.07.2018	23.08.2018	F	A	A3	52.56 N, 85.40 E	47	161	–
K5	KITE29	C 835594	10.07.2018	09.09.2019	F	A	A4	52.46 N, 85.14 E	426	692	–
K6	KITE31	C 835584	07.07.2018	14.09.2019	F	B1	B14	52.60 N, 85.27 E	434	963	a
K7	KITE32	C 835585	07.07.2018	13.09.2018	M	B1	B14	52.60 N, 85.27 E	68	265	a
K8	KITE33	C 835586	08.07.2018	05.08.2018	F	B1	B14	52.51 N, 85.36 E	28	148	–
K9	KITE34	C 835588	08.07.2018	21.10.2021*	M	B1	B6	52.51 N, 85.41 E	1024	2714	–
K10	KITE35	C 835589	09.07.2018	21.10.2021*	M	B1	B19.1	52.46 N, 85.23 E	1023	2239	–
K11	KITE36	C 835592	09.07.2018	03.12.2018	F	B1	B6	52.46 N, 85.14 E	147	305	b
K12	KITE37	C 835590	09.07.2018	28.08.2018	M	A	A3	52.44 N, 85.12 E	50	145	–
K13	KITE38	C 835593	09.07.2018	01.09.2018	M	B1	B6	52.46 N, 85.14 E	54	153	b
K14	OT-013	Not ringed	20.07.2019	08.06.2020	F	B1	B19	49.91 N, 88.99 E	324	1209	–
K15	OT-014	Not ringed	22.07.2019	16.09.2019	F	B1	B19	50.02 N, 89.13 E	56	56	–
K16	OT-015	Not ringed	20.07.2019	08.10.2020	M	B1	B19.1	49.91 N, 88.99 E	446	2789	–
K17	OT-016	Not ringed	23.07.2019	21.10.2021*	F	B1	B14	50.04 N, 89.16 E	645	61,645	–
K18	OT-017	Not ringed	26.07.2019	12.10.2021*	F	B1	B6	50.08 N, 89.04 E	641	85,946	c
K19	OT-018	Not ringed	02.08.2019	03.10.2019	F	B1	B6	50.08 N, 89.04 E	62	112	c

F, female; M, male; * birds were tracked also after 30 June 2021 (further data not included in this paper); aa, bb, cc, pairs of siblings.

### DNA examination

Contour pin feathers (newly grown feathers, full of blood) were collected from the lower part of a chick body and stored in 96% ethanol. The total DNA was isolated using the ExtractDNA Blood kit (Evrogene, Russia). The sex of tagged birds was determined by a method by Suh et al. (2011). A 699 bp fragment of the mitochondrial *cytB* gene was analysed to identify haplotypes^36^. The *cytB* mitochondrial gene fragment (924 bp) was amplified using F3 (5′-CCACCCCATCCTCAAAATAA-3′) and R8 (5′ATTGTGCGCTGTTTGGACTT-3′). We sequenced PCR fragments in both directions using a 3500 Genetic Analyser capillary sequencer (Applied Biosystems, USA) and aligned resulting sequences using the Vector NTI software (Thermo Fisher Scientific, USA). In order to exclude contamination, operations with genomic DNA and with PCR products were performed in different rooms. In unclear cases, PCR and sequencing were repeated.

### Satellite telemetry devices

Black Kites (*pulli*) were fitted with telemetry loggers in nests in 2018 (subpopulation A, Biysk) and 2019 (subpopulation B, Kosh-Agach). Loggers equipped with solar panels (20 g; Ecotone, Poland, and Ornitela, Lithuania; www.ecotone.pl, www.ornitela.com, respectively) were used to track the birds. Loggers were fitted onto the backs of the birds using harnesses (backpacks) consisting of a 6 mm Teflon ribbon encircling the body by two loops around the bases of the wings and joined in front of the breastbone. Loggers function in GPS (Global Position System)/GSM (Global System for Mobile Communication) systems. The GPS positions of the birds were collected according to individual settings (usually one position fixed per 1–6 h). They were sent as SMS (Short Message Service) text messages by local mobile operators to the Ecotone and Ornitela Centers in Poland and Lithuania, respectively, where they were saved and archived. To analyse the coordinates of bird positions and to create maps of migration we used GIS and the software ArcGIS 10.1 (Esri, Redlands, CA, USA).

### Data processing, migration characteristics

We processed positional data (coordinates) from studied birds for each bird individually. These data were separated into yearlong modules from 01.07.20XY (in the first year from the date of tagging) to 30.06.20XY + 1. The number of modules depends on the lifespan of each bird. We calculated the total distance travelled within the yearlong period and the number of temporary settlements areas (TSA) from these modules. We defined total distance travelled as distances between night roosting places connected chronologically (daily local movements were not calculated within the migration movement).

We defined TSA as a preferred place where a bird stayed for > 10 nights within 80 km^2^. This template size was based on roost locations distributed within a 10-km diameter over 10 days, thus, all falling within 80 km^2^^19^. Spring (pre-breeding) and autumn (post-breeding) migrations separate the winter and summer period. We defined the beginning of those migrations as a day when a bird left the winter/summer area and flew north/south without returning back in consecutive days. The end is defined as a day when a bird reached the summer or winter destination. Bird reached the summer or winter destination when it did not continue on its migration to north or south. During both migrations, birds tend to use stopovers, defined as a day with less than 50 km of a directed flight^26^. The size of the post-fledging area (PFA), winter and summer grounds (home ranges) between migrations were calculated as a Kernel density estimate (KDE) 95%. Before performing KDE estimation, we standardized the data set of each bird to 4 GPS fixes per day (1 each 6 h).

The Himalayas crossing was defined as the period of migration between the first and last day of migration with coordinates recorded by the foothill of the Himalayas with at least one coordinate recorded at over 5000 m asl. For this purpose, we set the loggers to collect the data every 5 min. During the crossing we defined active travelling hours of birds as the time between first and last coordinates with recorded speed over 5 km/h^37^. We classified manually and calculated the length of trajectory segments leading parallel with mountain ridges during the crossing and compared them with the overall distance travelled during the crossing over the Himalayas. We found segments parallel if the bird flew along a mountain slope copyrighting the valley and perpendicular if the bird flew across valleys and ridges not copyrighting the valleys.

We defined checkpoints W1, W2 and W3 as night positions where birds stayed on 31 January of their 2cy (second calendar year), 3cy and 4cy, respectively. It represents where birds were wintering during this date during the first, second and third winter. We defined checkpoints S1, S2 and S3 as positions where birds stayed during the breeding period on 30 June of their 2cy, 3cy and 4cy, respectively. We used the positions during S1, S2, S3, W1, W2 and W3 to compare the latitude of summer and winter areas used by individual birds between years of their life span and among individuals during the first, second and third years of their life.

### Meteorological data

Elevation data was downloaded from the mapping and analysing platform www.databasin.org “30 arc-second DEM of Asia” as a digital elevation model (DEM).

Weather data (wind, temperature and humidity) were obtained from the NCEP/DOE Reanalysis II dataset, using the RNCEP package^38^ for the R-software^39^. Weather data of crossing over the Himalayas were extracted for each coordinate in real-time, and pressure level of 700 hPa corresponding to an altitude between 2300 m and 3150 m. Airspeed, flow-assistance and absolute sidewind were calculated by function NCEP.tailwind using RNCEP package, which calculates flow-assistance and forward and sideways movement according to equation Tailwind (Tailwind = wind speed * cos (α), where α is the angle of the wind from the direction of travel). Equation Tailwind considers flow-assistance to be the component of the flow moving parallel to the specified direction (tailwind), with negative values indicating flows against the specified direction (headwind). We have extracted the weather data for coordinates recorded during post-breeding (n = 1790) and pre-breeding (n = 1310) migration over the Himalayas. We excluded coordinates recorded while roosting from the dataset (coordinates with recorded speed lower then 5 km/h).

### Statistical analysis

We performed the Mann–Whitney U test for testing the differences in pre-breeding and post-breeding migration and home-range characteristics and the pre-breeding and post-breeding Himalaya’s flight-over characteristics. To assess the difference in total distance travelled, number of TSA, and the size of home ranges in summer and winter quarters over the years, we performed Kruskal–Wallis ANOVA test. Before any statistical comparison, we run the Shapiro–Wilk test for normality to assess the data distribution. To assess the effect of weather on bird’s movement across the Himalayas, we used linear mixed models (LMMs) in R software using the ‘lme4’ package^40^ to analyse the following dependent variables: bird groundspeed and airspeed, in relation to season, flow-assistance, sidewind, humidity and temperature during the crossing over the Himalayas (Table 2). We used LMM with bird ID as a random effect (as individuals could be tracked over multiple years). Only birds with telemetry loggers Ornitela, which crossed over the Himalayas, were included in the LMM (K14 – K19) due to the high frequency of coordinates recording. The best supported LMM model was selected according to the lowest Akaike's information criterion for a small sample size (AICc)^41^. All statistical tests were performed using an α-value of 5%, and all mean values are presented (± standard deviation; SD) unless stated otherwise.

**Table 2:** Selecting the best LMMs for the airspeed and groundspeed during the Himalayas crossing. We listed first six models for each dependent variable.

Dependent variable	Explanatory variable	Df	AIC	ΔAIC	R^2^
Airspeed	~ TW + SW + Seas	2724	16755	0	0.52
	~ TW + SW + Seas + Temp + Hum	2723	16764	9	0.14
	~ TW + SW + Seas + Temp	2849	17541	786	0.19
	~ TW + SW	2850	17544	789	0.1
	~ Seas	2851	17557	802	0.2
Groundspeed	~ TW + SW + Seas	2724	15859	0	0.42
	~ TW + SW + Seas + Temp + Hum	2723	15869	10	0.34
	~ TW + SW + Seas + Temp	2725	15908	49	0.3
	~ TW + SW	2850	16712	853	0.29
	~ TW + SW + Temp	2849	16714	855	0.25
	~ Seas	2851	17021	1162	0.13

Models are ranked according to increasing ΔAIC values, with the best performing model on top. TW—tailwind; SW—sidewind; Seas—season; Temp—temperature; Hum—humidity.

## Results

### *cytB* haplotypes

Black Kites from Biysk belonged to haplogroup A (haplotypes A3 and A4) and haplogroup B (haplotypes B6, B6.1, B14, B19 and B19.1). Three families and 8 families had haplogroups A and B, respectively (Table 1). All Black Kites (5 families) from Kosh-Agach belonged to haplogroup B (haplotypes B6, B14, B19 and B19.1).

### Migration routes and total distance travelled

**Figure 1:**
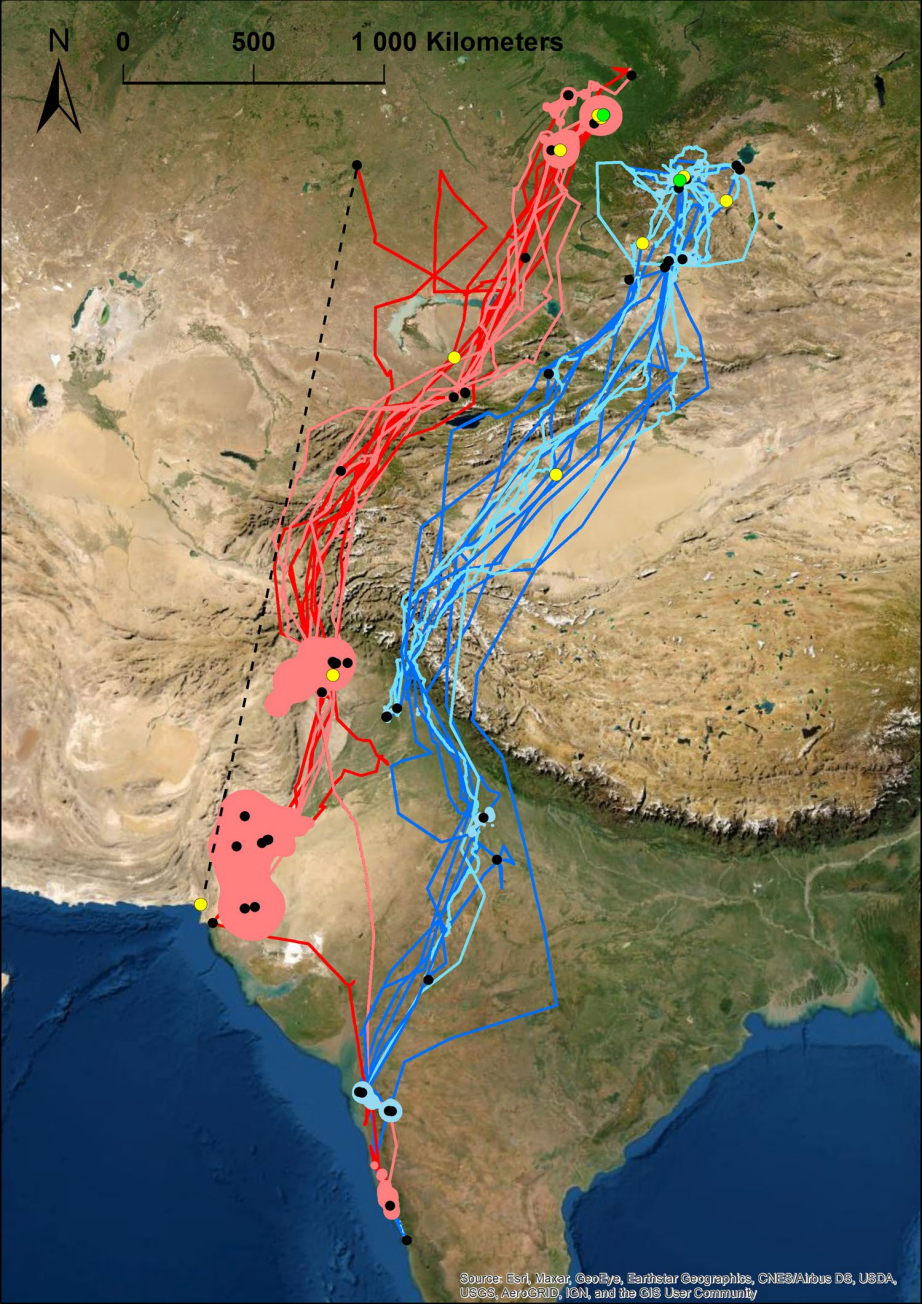
Migration routes and pre-breeding and post-breeding home ranges of Black Kites tagged in Biysk (dark red lines—autumn migration, light red lines—spring migration, light red polygon—homerange) and Kosh-Agach (dark blue lines—autumn migration, light blue lines –spring migration, light blue polygon—homerange). Black dots represent temporary settlement areas. Green dots represent natal nests, yellow dots represent last recorded positions. Black dashed line represents the link between positions of K5 before and after a gap in data collection. Figure was created using software ArcGIS 10.1 (Esri, Redlands, CA, USA). Map source: Esri, Maxar, Earthstar Geographics, USDA FSA, USGS, Aerogrid, IGN, IGP, and the GIS User Community, "Wolrd Imagery", December 12, 2012. https://www.arcgis.com/home/item.html?id=10df2279f9684e4a9f6a7f08febac2a9. Accessed on October 28. 2021.

Black Kites originating from Biysk migrated through the Western Circum-Himalayan Corridor (Fig. 1). These birds flew through eastern Kazakhstan, Kyrgyzstan, Tajikistan and eastern Afghanistan to winter, mainly in northern and southern Pakistan and western India. After winter, birds flew over the same migration corridor back to Biysk area. Unlike Kites from Biysk, Black Kites originating from Kosh-Agach used a different migration route (Fig. 1). These birds flew over Tian Shan, and the Taklamakan Desert in China, followed by Trans-Karakoram crossing-over through Jammu and Kashmir to winter in northern and western India and eastern Pakistan. After winter, birds flew over the same corridor back to Kosh-Agach area.

The average total distance travelled of birds from both subpopulations in the first year was 9191 km (ranging from 6431 to 12,478 km). During the first year, birds used on average 4 TSAs (ranging from 2 to 6). During the second year, birds travelled on an average total distance of 9121 km (ranging from 7422 to 11,268 km) using 5 TSAs (ranging from 4 to 7). The average total distance travelled in the third year was 6839 (ranging from 6594 to 7084) using 5 TSA (ranging from 4 to 5) (Table 3).

**Table 3:** Characteristics of the total distance traveled during a one year long modul.

	1st year		2nd year		3rd year	
	Trajectory (km)	TSA	Trajectory (km)	TSA	Trajectory (km)	TSA
K3	9049	2	11,268	7	-	–
K5	9883	3	–	–	–	–
K6	12,478	3	–	–	–	–
K9	6431	2	7422	5	7084	5
K10	7113	5	8034	5	6594	4
K14	10,031	4	–	–	–	–
K16	11,070	4	–	–	–	–
K17	7566	4	8827	4	–	–
K18	9097	6	10,055	6	–	–
**Mean**	9191	4	9121	5	6839	5
**SD**	1940	1	1140	1	346	1

Only birds that survive for at least one year (from the date of tagging untill the 30. 06. following year) are included in the table.

Five tagged birds survived and were tracked for multiple years. For those individuals, we compared the differences in the total distance travelled and the number of used TSAs. We found no significant difference in the total distance travelled (P > 0,05) nor the number of TSA (P > 0,05) used among the years. We also tested the difference in total distance travelled, and the number of TSA used between the two subpopulations without any significant results (P > 0,05).

### Timing of autumn and spring migrations

Timing of autumn migration varied slightly among individuals in departure date (30 August ± 12 days) and noticeable more in arrival date (26 October ± 84 days). The timing of spring migration also varied slightly in departure date (17 April ± 12 days) and arrival date (09 May ± 14 days). Surprisingly, timing of either migration did not vary along different age groups. The tagged kites travelled relatively fast, completing 2535–4842 km journey in 10–94 days, progressing by 62–253 km/day, with significantly faster speeds and lower need to rest in the pre-breeding migration (Table 4). During the pre-breeding migration was the speed and active speed more than 50% and 30% higher in comparison to post-breeding migration. As a result, the pre-breeding migration lasted 10 days less.

**Table 4:** Estimates of post and pre-breeding migration by tagged Black Kites from 2018 to 2021.

**Migration component**	**N**^**a**^	**Post-breeding migration Mean ± SD**	**N**^**a**^	**Pre-breeding migration Mean ± SD**	**U**	**P**
Departure date^b^	22 (11)	240 ± 12	16 (9)	107 ± 12		
Arrival date^b^	22 (11)	293 ± 84	16 (9)	129 ± 14		
Speed (km/day)	22 (11)	106 ± 56	16 (9)	165 ± 60	60	**0.00**
Active speed (km/travelling days)	22 (11)	129 ± 55	16 (9)	162 ± 62	79	**0.04**
Duration (days)	22 (11)	36 ± 11	16 (9)	23 ± 9	86	**0.03**
Travelling days	22 (11)	21 ± 15	16 (9)	22 ± 9	133	0.9
No. stopovers	22 (11)	0.63 ± 0.8	16 (9)	0.3 ± 0.2	112	0.28
Days of stopover	22 (11)	9 ± 14	16 (9)	1 ± 3	83	**0.03**
Route length (km)	22 (11)	2874 ± 764	16 (9)	3279 ± 769	96	0.09

^a^Number of migration episodes (number of tagged individuals). ^b^Julian date (1 = 1 January). Birds that died before or during migration were omitted from the test. Differences in mean values were tested by Mann–Whitney U test for nonparametric data and significant results are highlighted in bold.

### Post-fledging area and home ranges in winter and summer

**Figure 2:**
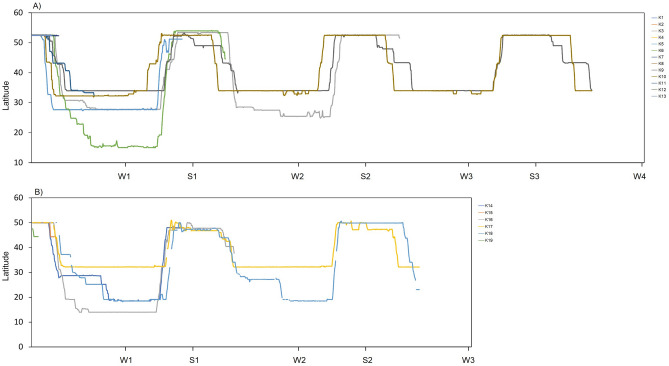
Latitudinal occurrences of tracked Black Kites throughout their lifespan. W1, W2 and W3 refer to the location of birds on 31 January in 2cy, 3cy and 4cy, respectively; S1, S2 and S3 refer to the location of birds on 30 June in 2cy, 3cy and 4cy, respectively. (**A**) Black Kites from Biysk (lowland in southwestern Siberia); (**B**) Black Kites from Kosh-Agach (Upper Altai).

**Figure 3:**
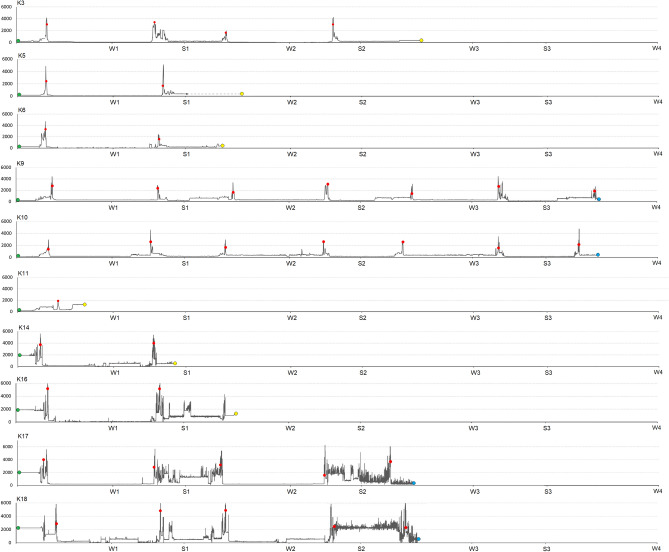
Elevation profile of lifelong journeys of tracked Black Kites. Green dots represent the nest, red dots represent highest roost points during migrations, yellow dots represent the last position collected due to the death of birds or signal loss, blue dots represent the last position collected of living birds. For latitudes in W1, W2, W3 and S1, S2, S3 see Fig. 2.

**Figure 4:**
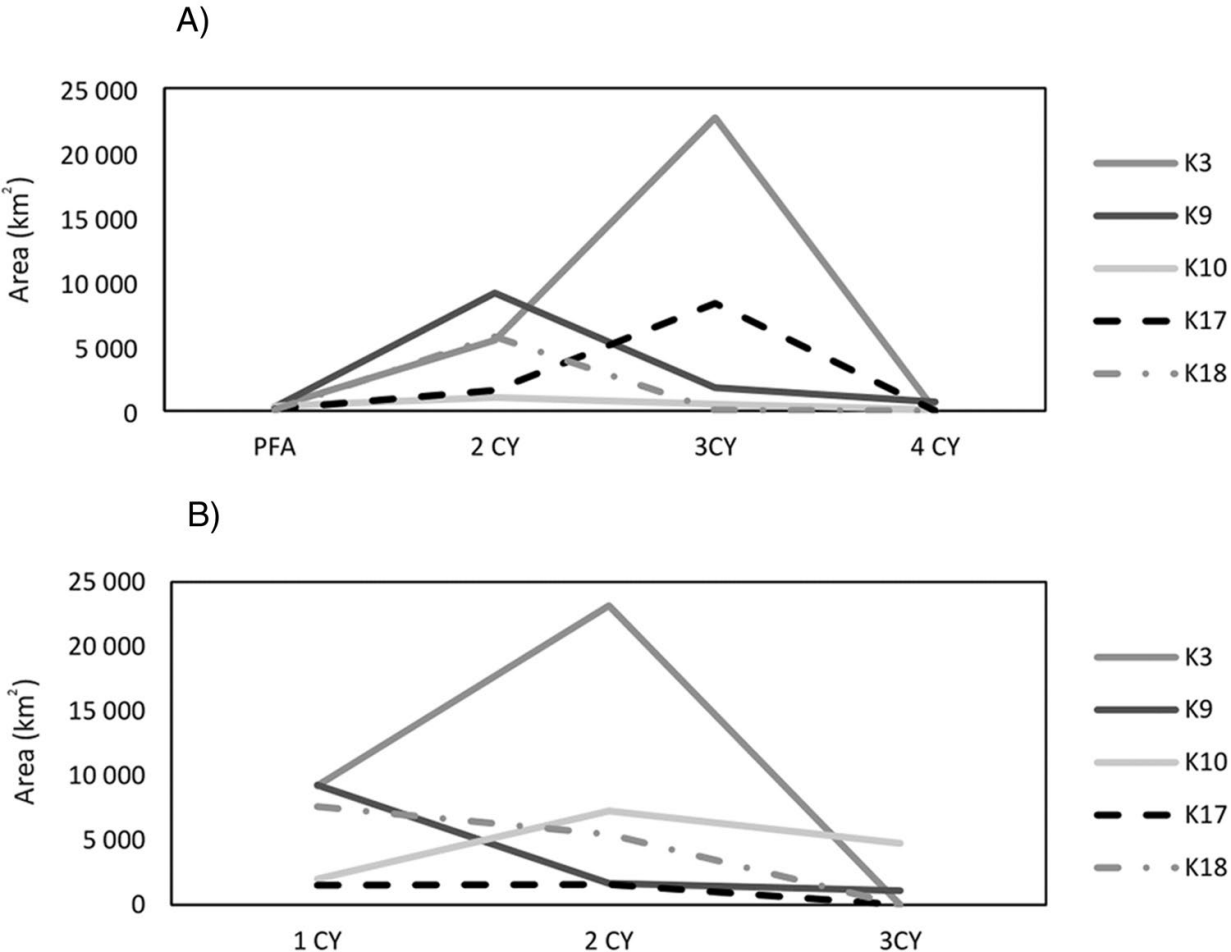
Line chart showing the changes in the size of (**A**) summer and (**B**) winter home-range throughout the life span of five tagged Black Kites. PFA—post fledging area; CY—calendar year.

The post-fledging area of tagged Kites varied from 1.7 km^2^ to 1567 km^2^ with a mean of 396 ± 432 km^2^ (Table 5). Some birds left the nest and flew straightforward to the winter quarters. Others birds explored the area around the nest and departed for autumn migration with a slight delay. Black Kites from both subpopulations wintered in Indian Subcontinent in low elevations of areas with high human footprint in Pakistan and India (Figs. 1 and 2). No bird remained in the Indian subcontinent during summer periods (Fig. 2). Birds wintered on average for 190 days, and the mean area of individual home ranges was 4704 km^2^ (Table 5). During the breeding period, birds occupied areas in south-western Siberia, where they spent on average 125 days with an average home range size 3537 km^2^ (Figs. 1 and 2; Table 5). No bird remained in Siberia during the winter period (Fig. 2). Although the mean area of home ranges was slightly smaller during the breeding season than in the nonbreeding winter period, we found no statistical difference in the spatial use (p > 0.05). Five tagged Black Kites survived and were tracked for multiple years (Fig. 3). For those individuals, we compared the differences in the area size of home ranges in the breeding (summer quarters) and nonbreeding season (winter quarters). We found no difference in spatial use over the years in neither the winter quarters (p > 0.05) or summer quarters (p > 0.05). Birds showed individual changes in the size of winter and summer home-range over the course of time (Fig. 4).

**Table 5:** Characteristics of post fledging area (PFA), winter and summer quaters. Kernel density etimate (KDE) are stated in km^2^.

	**PFA**		**1. winter**		**1. summer**		**2. winter**		**2. summer**		**3. winter**		**3. summer**	
	**Days**	**KDE 95%**	**Days**	**KDE 95%**	**Days**	**KDE 95%**	**Days**	**KDE 95%**	**Days**	**KDE 95%**	**Days**	**KDE 95%**	**Days**	**KDE 95%**
K1	22	59	–	–	–	–	–	–	–	–	–	–	–	–
K2	38	174	–	–	–	–	–	–	–	–	–	–	–	–
K3	60	273	204	9214	138	5482	201	23,167	125	22,708	–	–	–	–
K4	47	1349	–	–	–	–	–	–	–	–	–	–	–	–
K5	49	501	222	980	44	480	–	–	–	–	–	–	–	–
K6	60	454	170	6849	103	439	–	–	–	–	–	–	–	–
K7	68	539	–	–	–	–	–	–	–	–	–	–	–	–
K8	28	17	–	–	–	–	–	–	–	–	–	–	–	–
K9	52	295	211	9278	101	9129	184	1681	121	1791	172	1101	91	694
K10	47	369	205	2002	109	1040	198	7272	132	519	188	4762	145	93
K11	46	552	–	–	–	–	–	–	–	–	–	–	–	–
K12	50	1576	–	–	–	–	–	–	–	–	–	–	–	–
K13	54	515	–	–	–	–	–	–	–	–	–	–	–	–
K14	35	1.7	213	2853	–	–	–	–	–	–	–	–	–	–
K15	45	4	–	–	–	–	–	–	–	–	–	–	–	–
K16	56	606	173	157	127	1779	–	–	–	–	–	–	–	–
K17	44	147	221	1518	68	1596	172	1584	119	8327	–	–	–	–
K18	50	84	134	7611	95	5741	185	5475	126	65	–	–	–	–
K19	47	8	–	–	–	–	–	–	–	–	–	–	–	–
**Mean**	47	396	195	3474	98	3211	194	7707	125	6682	180	2931	118	393
**SD**	11.0	433	30	3475	30	3187	9	5147	5	9556	11	2508	37	324

### High-elevation crossing of the Himalayas and influence of the wind on the crossing over the Himalayas

Timing of post-breeding and pre-breeding crossing over Himalayas varied slightly among individuals in departure dates (20 September ± 12 days; 28 April ± 7 days) and arrival dates (21 September ± 12 days; 29 April ± 7 days). Black Kites originating from Koch-Agar travelled relatively fast, crossing over the Himalayas (on average 571 km) in 2 days, progressing with average active speed of 30.2 km per travelling hour, flying from 6 to 10 h per day. Active speed and number of traveling hours were slightly higher during pre-breeding flight-over. During the crossing of the Himalayas birds roosted for one night in average altitude of 4589 m asl, ranging from 1577 to 5171 m asl (Table 6, Fig. 3).

**Table 6:** Estimates of post and pre-breeding migration over the Himalayas by tagged Black Kites during 2019 and 2021.

Migration component	N^a^	Post-breeding migration Mean ± SD	N^a^	Pre-breeding migration Mean ± SD	U	P
Departure date^b^	9(4)	260 ± 12	6(4)	118 ± 7		
Arrival date^b^	9(4)	261 ± 12	6(4)	119 ± 7		
Duration (days)	9(4)	2	6(4)	2		
Active speed (km/travelling hours)	9(4)	30.2 ± 3.1	6(4)	32.7 ± 4.3	36	0.08
Travelling hours per day	9(4)	8.6 ± 1.4	6(4)	8.9 ± 1.2	52	0.50
Route length (km)	9(4)	569 ± 71	6(4)	572 ± 54	18	0.58
PFAR (%)	9(4)	29.5 ± 2.6	6(4)	55.5 ± 15	26	**0.00**
Highest altitude	9(4)	5842 ± 149	6(4)	5978 ± 309	7	0.07
Roost altitude	9(4)	4589 ± 781	6(4)	3827 ± 1938	10	0.26
Airspeed (m/s)	9(4)	7.1 ± 5.1	6(4)	9.2 ± 6	542	0.16
Groundspeed (m/s)	9(4)	7.9 ± 4.4	6(4)	12.3 ± 6	397	**0.00**
Flow-assistance (m/s)	9(4)	-1.8 ± 2	6(4)	0.8 ± 2	150	**0.00**
Sidewind (m/s)	9(4)	0.6 ± 2.3	6(4)	0.9 ± 4.2	449	**0.00**
Temperature (°C)	9(4)	9 ± 2	6(4)	5 ± 2	145	**0.00**
Relative humidity (%)	9(4)	71 ± 12	6(4)	63 ± 15	402	**0.00**

^a^Number of migration episodes (number of tagged individuals). ^b^Julian date (1 = 1 January). Departure date—first day with coordinates recorded in altitude over 5000 m above the sea level, Arrival date—last day with coordinates recorded in altitude over 5000 m above the sea level, Airspeed—speed of a bird relative to the air. Groundspeed—speed of bird relative to the earth. PFAR—percentage of parallel flight along the mountain ridge. Only birds K14–K19 were included in the table due to high frequency of data collection. Birds that died before or during migration were omitted from the test. Differences in mean values were tested by Mann–Whitney U test for nonparametric data and significant results are highlighted in bold.

**Figure 5:**
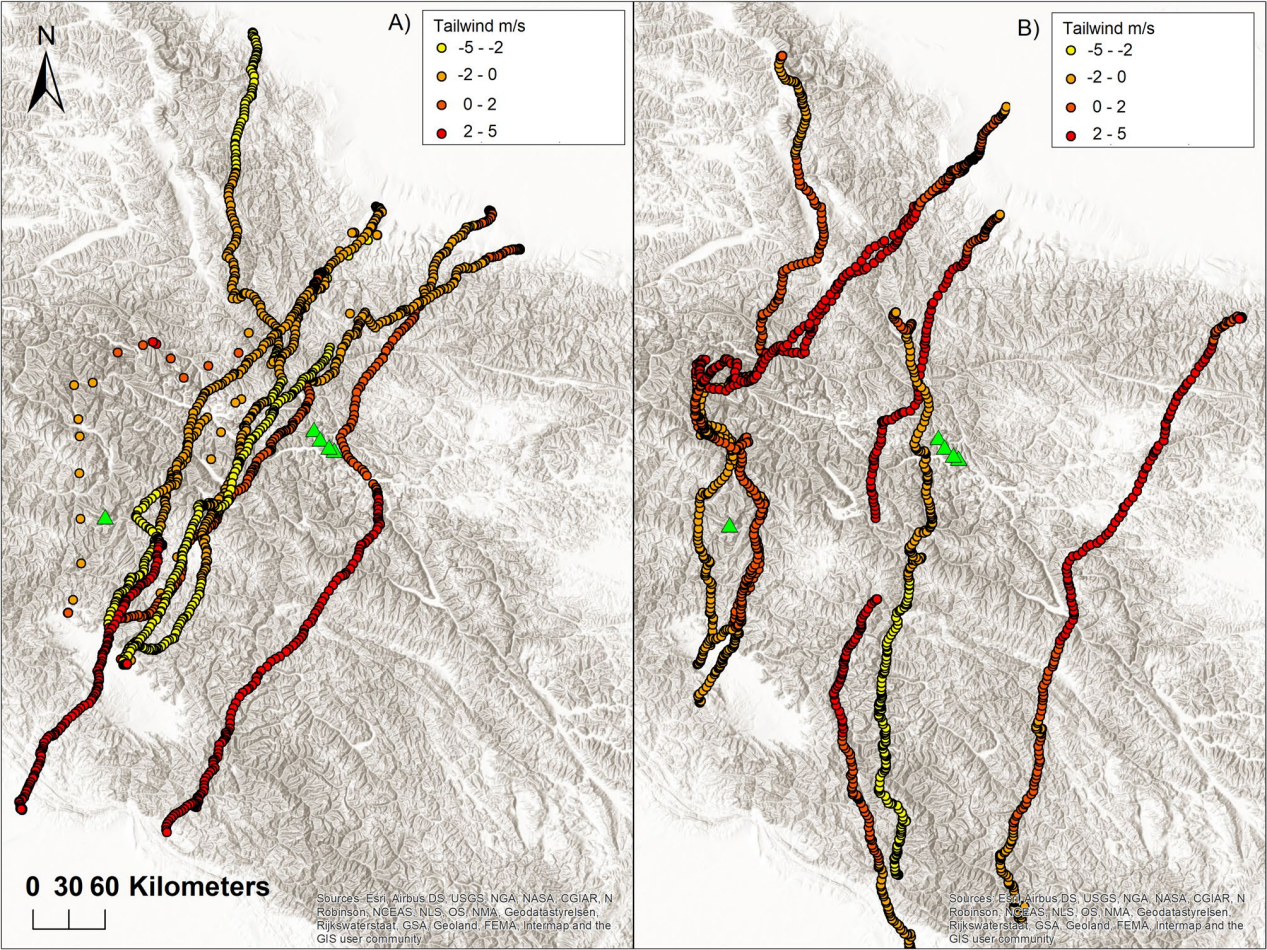
Post-breeding (**A**) and pre-breeding (**B**) crossing over the Himalayas in relation to tailwind speed. Green triangles represent mountain peaks over 8000 m. Figure was created using software ArcGIS 10.1 (Esri, Redlands, CA, USA). Map source: Esri, USGS, Airbus DS, NGA, NASA, CGIAR, N Robinson, NCEAS, NLS, OS, NMA, Geodatastyrelsen, Rijkswaterstaat, GSA, Geoland, FEMA, Intermap, and the GIS user community, "Wolrd Hillshade", October 18. 2018. https://www.arcgis.com/home/item.html?id=babedc22ebd64a428b77f7119c2591c3. Accessed on October 26. 2021.

Wind condition significantly varied during the pre-breeding and post-breeding Himalaya flight-overs (Table 6). Noticeable was the difference in the tailwind speed, sidewind speed and percentage of parallel flight along the mountain ridge. While during the post-breeding flight-over, birds faced mostly a headwind and preferred to fly perpendicularly to mountain ridges and mountain valleys, on their pre-breeding flight-over, birds flew with a tailwind and preferred to fly parallelly along the mountain ridges and mountain valleys (Fig. 5).

**Figure 6:**
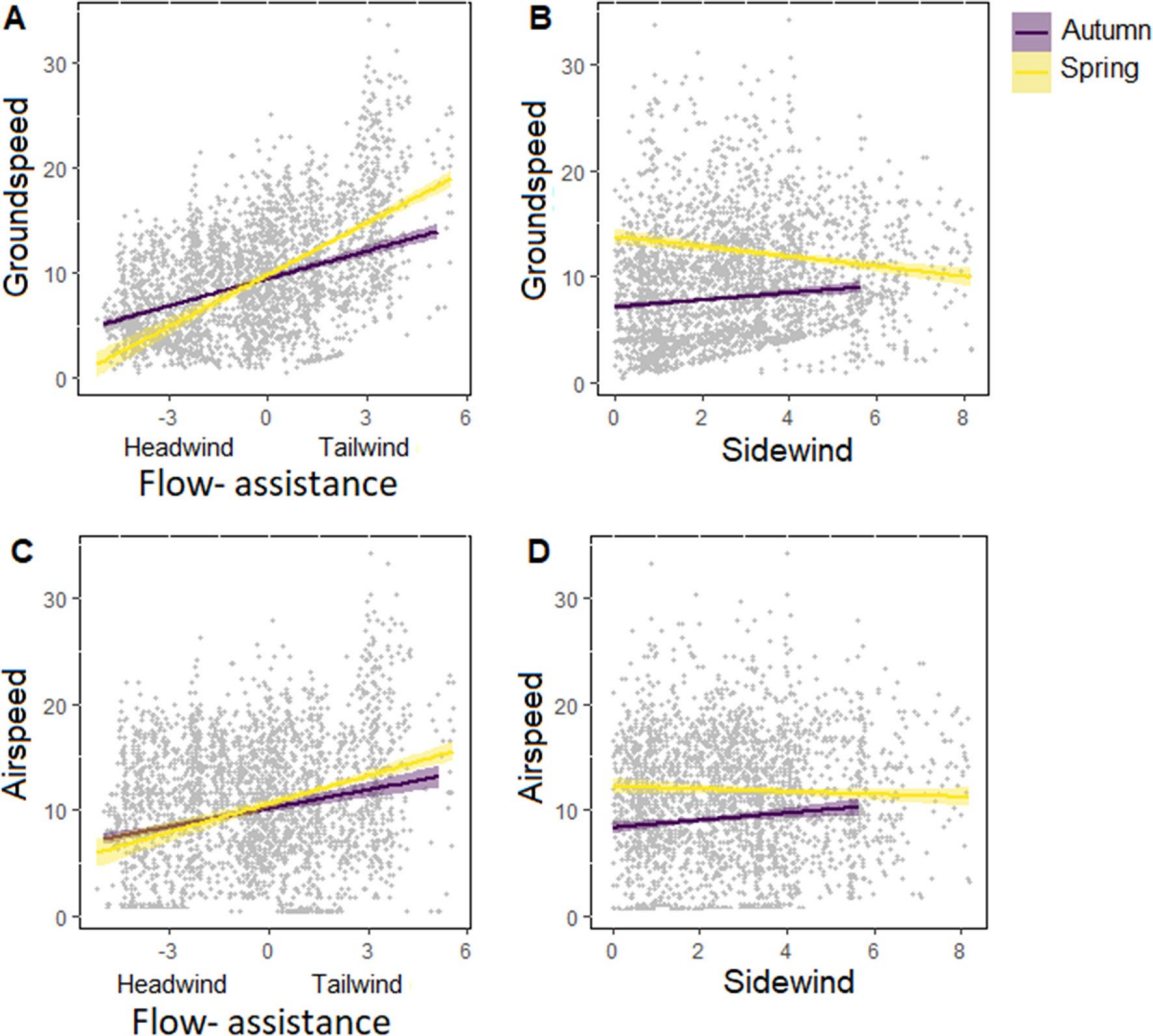
Black Kites groundspeed and airspeed fitted with linear regression lines (colorful lines, with 95% CI as the same colour area) in relation to sidewind and flow-assistance during the autumn/post-breeding (purple) and spring/pre-breeding (yellow) crossing over the Himalayas.

Our best LMM model showed that airspeed of birds crossing over the Himalayas were not only positively related to tailwind but also to difference in season. The groundspeed was also positively affected by tailwind and season but negatively affected by sidewind (Table 7). Although the model results showed negative effect of sidewind to groundspeed, plotting the linear regression lines by season showed that the sidewind had a slightly positive effect on groundspeed during autumn migration (Fig. 6B,D). Prevailing tailwind had generally greater positive effect on both the air- and groundspeed of bird during the spring migration (Fig. 6A,C).

**Table 7:** Fixed effects on bird groundspeed and airspeed as estimated by our best LMMs. We consider coefficient estimates to be significant at P < 0.05 (bold).

Dependant variable	Explanatory variable	Estimates	SE	t	Pr( >|t|)
Airspeed	(interpcet)	4.67	1.13	4.12	**0.12**
	Tailwind	0.20	0.06	3.39	**0.00**
	Sidewind	0.03	0.07	0.36	0.71
	Season_spring	2.71	0.4	6.61	**0.00**
Groundspeed	(interpcet)	6.29	0.56	11.1	**0.00**
	Tailwind	0.89	0.05	17.2	**0.00**
	Sidewind	-0.13	0.06	4.84	**0.00**
	Season_spring	2.48	0.34	7.17	**0.00**

## Discussion

### Subspecies status of examined Black Kites and their migration routes

We found different sets of *cytB* haplotypes in Black Kites from Biysk (Altai Krai) and Black Kites from Kosh-Agach (Altai Republic). While in Kosh-Agach, the haplotypes were characteristic of *M. m. lineatus*, in the vicinity of Biysk, the haplotypes were characteristic for both subspecies *M. m. migrans* and *M. m. lineatus,* indicating a hybrid population from the intergradation zone between *M. m. migrans* and *M. m. lineatus*^36,42^. Lindholm and Forsten^43^ were aimed at subspecies determination of Black Kites in Altai Krai and Altai Republic according to morphological features^16^. They found that birds in the lowlands of Altai Krai, on average, were different from those in the higher country of the Altai Republic. Black Kites from the lowlands of Altai Krai had some features of *M. m. migrans* (i.e., birds originated from the intergradation zone between *M. m. migrans* and *M. m. lineatus*). Black Kites from Altai Republic were quite similar to the easternmost typical *lineatus* and were placed in that taxon (i.e., *M. m. lineatus*). This conclusion fits well with our observations, and it is consistent with our *cytB* haplotype results. We consider Black Kites tagged in Byisk as birds from the intergradation zone between *M. m. migrans* and *M. m. lineatus* and Black Kites tagged in Kosh-Agach as birds belonging to *M. m. lineatus*. Deep genomic study of Black Kites from various parts of their breeding area is needed to solve the genetic structure of their populations and hence, their species/subspecies status.

As we mentioned earlier, raptors of several species were observed to migrate across the Himalayan region throughout four main corridors: (1) Western Circum-Himalayan Corridor, (2) Eastern Circum-Himalayan Corridor, (3) East-to-West Southern Corridor and (4) Trans-Himalayan Corridor^27^. We demonstrated that Black kites from Biysk (birds from intergradation zone between *M. m. migrans* and *M. m. lineatus*) used Western Circum-Himalayan Corridor and Black Kites from Kosh-Agach (*M. m. lineatus*) used Trans-Himalayan Corridor. Although there is not large distance between Black Kites from Biysk and Kosh-Agach, birds from these populations chose narrow and non-overlapping migration corridors, where one involved crossing of the Himalayas. Wintering ranges of birds from these two populations where also distanced and non-overlapping. The only interaction between birds from these populations was found in one bird from Biysk which shared the winter area with birds from Kosh-Agach. We assume that the genetic background of the migration behaviour of Black Kites may be strong, forming the uniform behaviour of tracked birds from both studied subpopulations. What is more, our result showing a different genetic history of both populations supports our assumption.

On the other hand, studies showed that timing of migration does not always vary between young birds and experienced adults^17,44^. Our results also showed no difference in timing or route selection connected with different age. Under such scenario, young birds may migrate along experienced adults. This offers a social learning opportunity for young birds that can be hard to distinguish from genetic determination of migratory routes. Furthermore, variable routes of raptors (Peregrines, *Falco peregrinus*) migrating from Siberia to South Asia were demonstrated even if they had the exact gene involved in regulation of the migration distance^45^. Although we provide data showing differences in haplotypes between subpopulations, that might have influenced the uniformity within subpopulations, the role of innate factors and social learning in transmission of routes remain unclear and asks for further research.

### Bird (including Black Kite) migration over high altitudes in the Himalayas

High-altitude flights of birds over the Himalayas are a highly challenging feat of performance underpinned by several specialised physiological traits. Flapping birds like Bar-headed Goose and Ruddy Shelduck (*Tadorna ferruginea*) can reach high altitudes during their migration across the Himalayas and Tibetian plateau because they can support the metabolic costs of flight as the low-density air becomes extremely hypoxic^35,46^. Like other migrating (soaring) birds, they may occasionally use updraft wind assistance to help offset flight cost^47^. However, they experience periods of intense flapping flight that require extremely high heart rates, wing-beat frequencies, and metabolic power, such as during level flight at high elevation or during climbs that are not assisted by wind^35,48^.

Raptors use primarily soaring-gliding flight during migration^49^. Soaring flight is an energetically efficient form of flight, and many long-distance migrants are so-called obligate soaring migrants^27^. Updraught necessary for soaring flight includes thermals (pockets of warm rising air) and deflection (orographic) updraughts that occur when horizontal winds strike surface discontinuities, including mountains. The high-altitude terrain of the Himalayas precludes this type of pathway, and hence it is used by raptors^27^. However, some raptors, especially falcons, use flapping flight on their migration across the Himalayas^45^.

Unfortunately, detailed studies using telemetry devices on raptors crossing the Himalayas are scarce. We can compare our results mainly with a recent study aimed at Black Kites fitted with telemetry loggers in Dehli, India^26^. It seems that Black Kites tagged in Dehli originated, similarly like in our study, to two different population: birds that used Western Circum-Himalayan Corridor may belong to Black kites originating from the intergradation zone between *M. m. migrans* and *M. m. lineatus*, birds that used Trans-Himalayan Corridor may belong to *M. m. lineatus*. Migration routes of these birds were distinct in our study as well as like in a study by Kumar et al.^26^. The birds originating from Upper Altai (Kosh-Agach) crossed the Himalayas over Tian Shan Mts, Taklamakan Desert, and Karakoram Mts like the main portion of Black Kites tagged in Dehli. These birds crossed the Himalayas in extremely high elevation up to 6281 m asl and travelled long periods at elevations above 3500 m. Birds flew across the Himalayas for two days with a single stop to roost at elevations between 1644 to 5448 m asl.

Black Kites crossing the Himalayas may have physiological adaptations that remain to be investigated. They fly and, moreover, stay for hours resting at night in the environment of mountains at altitudes over 5000 m with variable wind speed and direction, where the air density and partial pressure of oxygen is roughly half of that at sea level^35,50^. At the same time, the temperature can be very low, well below freezing year-round, which could require additional metabolic energy for thermogenesis. Maintaining water balance during flight should also be a major challenge in the dry air at high altitudes^35,50^.

### Ontogenetic shifts in summer areas of immature Black Kites

Contrary to immature Black Kites using the West African-Eurasian flyway^19^, immature Kites in our study returned to the natal area in their first years of life or migrated to even more northerly areas. It seems that high behavioural flexibility is apparent during summer stays of immature Black Kites. Furthermore, such a difference in behaviour brings up an assumption that birds crossing over Himalayas are less constricted by barriers then those wintering in sub-Sahara part of Africa. We believe that the reason for such a difference may be more complex conditioned by many factors such as climate, habitat quality, food abundance, density of populations in breeding areas and with it connected competition and possibly genetic background. Although our results showed that ontogenetic shifts may differ between subspecies of a single species, the causes and consequences of such a variation remain unknown and require further research. Unlike Kumar et al.^26^, we found no difference in the size of the home range during the breeding and nonbreeding seasons.

### Environmental influence on migration

Route configuration of Black Kites crossing the Himalayas seemed to be shaped by dominant wind support and barrier avoidance^26^. Black Kites perform circular soaring in areas of higher predicted thermal uplift and linear soaring in areas of higher predicted orographic uplift velocity^51^. During the pre-breeding crossing over Himalayas birds tent to fly parallelly along with the mountain ranges, through the mountain valleys using the up-lifting anabatic winds for soaring up to high altitudes and gliding with the possible strong south valley tailwinds^52^. During the period of pre-breeding migration (from the end of April to the beginning of May, which correspond to the timing of spring migration of tagged Black Kites) with the warmest and driest surface condition, great ascending thermals are forming, creating a great opportunity for soaring birds to glide over Himalayas^53^. While flying north along the mountain ridges, sidewind, that mostly blows from the west^52^, can break over the ridge creating a lee wind perpendicular to bird direction, that may have a negative effect on the birds' groundspeed as the bird has to angle towards the sidewind (as shown by our results). In contrast with the pre-breeding crossing, during the post-breeding Himalayas crossing over bird tent to fly directly across the mountain ranges.

We assume that birds used thermals to stay as high as possible to glid along or against the lee winds to avoid the strong headwinds of the valley breeze^52^. We found that Black Kites increased more their groundspeed and less their airspeed when tailwinds prevailed. For soaring migrants, reducing airspeed under tailwinds allows the birds to attain low sink rate and by that to cover larger distances while decreasing the risk of reaching the ground or switching to energy-expensive flapping flight^54^. However, during pre-breeding Himalayas crossing, birds noticeable increased their airspeed even during stronger tailwind. We believe that this behaviour is partly caused by the abundance of great ascending thermals. Bird can afford to increase its airspeed on the expense of higher sink rate in order to quickly pass the Himalaya barrier. Similar behaviour was observed in Honey Buzzards (*Pernis apivorus*) that were found to glide at fast airspeeds only in those areas where the best soaring conditions occurred^55^.

What we found interesting is the effect of different season on air and groundspeed of migrating birds. Tagged Kites kept increasing their airspeed even with prevailing tailwind, which shows on birds own motivation to increase its overall speed during the spring crossing on the expense of energy that they could have saved with lowering the airspeed in tailwind and gliding with low sink ratio. Many studies of avian migration showed that birds tend to migrate faster during spring migration than autumn migration^56^. Migration theory predicts that migrants minimalize the duration of spring migration to arrive in breeding area as soon as possible. Birds that arrive sooner start to breed earlier which can positively affect the reproductive performance^56,57^. Additionally, they will have more time for raising better quality offsprings that have better chance to survive their first migration^58^. Although there are cases when the spring migration took approximately the same time or longer^59,60^. We found the spring migration to be significantly shorter in comparison with autumn (post-breeding) migration, although the duration of the Himalayan crossing was found to be the same. As we mentioned earlier, for aerial migrants, wind represent a major support that can considerably reduce both energy and time cost of migration^61^. A stronger tailwind prevailing during spring increased the birds' speed and eased the Himalaya crossing. Birds were less exhausted from the Himalayas crossing over and arrived at summer destination much faster. Based on all that, we suggest that birds in our study migrated faster during the spring migration due to both favourable wind conditions and inner motivation.

## High behavioural flexibility of Black Kites to surmount environmental obstacles

The challenging environmental obstacles for Black Kites *M. m. migrans* migrating to winter in Africa are crossing large water bodies and desert^18^. Most migrating Black Kites *M. m. migrans* are reluctant to fly over large water bodies and cross transcontinental boundaries^6,19,62^. It seems that the vast breeding territory of Black Kites in the Palearctic realm is connected with the unusual behavioural flexibility of Black Kites to surmount various environmental obstacles on their migration routes.

This high behavioural flexibility may also elucidate a new important wintering area for Black Kites in the Middle East. Black Kites with *M. m. lineatus* features were recorded for the first time in the Levant area in Syria (and perhaps also in Lebanon) during the beginning of the second half of the twentieth century^63,64^. Novel observations of the communal roosting of Black Kites during the winter months have been reported in south-eastern Europe, Egypt, and Turkey; however, their taxonomic subspecies status was not mainly investigated^65–70^.

Increasing number of Black Kites spotted in the Middle East seems to be related to a consistent increase in Black Kites numbers migrating along eastern part of Black Sea from 2011^43^. Now, the Black Kite is the most common wintering raptor in Israel, and a proportion of kites wintering in Israel showed morphological characteristics of *M. m. lineatus*, likely representing the western outpost of wintering *M. m. lineatus*^71^. Alternatively, these individuals may comprise birds from the broad intergradation zone between *M. m. migrans* and *M. m. lineatus*^72^.

It now appears that Black Kites with *M. m. lineatus* features supposedly originated from a large intergradation zone between *M. m. migrans* and *M. m. line*atus can be found anywhere in Europe west of Russia^16^. Recent data on numerous wintering of Black Kites in Georgia in an area of the Black Sea Basin correspond well with these data^73^. Moreover, Black Kites with *M. m. lineatus* features can be found migrating from southern and eastern Africa as documented in South Africa in November 1972 and Ethiopia in November 2011^74,75^.

## Conclusion

By telemetry research and DNA analyse of Black Kites from Western Siberia we found differences in subpopulations of Black Kites from Upper Altai close to Kosh-Agach and Black Kites from Biysk, pointing at the intergradation zone between *M. m. migrans* and *M. m. lineatus* and revealing their migration routes. Black Kites *M. m. lineatus* migrating to winter in Indian Subcontinent were challenged by the crossing of the main Himalayan ridge. They flew and roosted in the environment of mountains at altitudes over 5000 m in unfavourable weather conditions. During crossing, birds showed a response to the wind direction which helped them to overcome the environmental obstacle. Remarkable behavioural flexibility of Black Kites to surmount various environmental obstacles on their migration routes may be one reason that the species has been able to colonize such a large breeding range and may also elucidate the ongoing rapid establishment of novel wintering areas by Black Kites. What is more, Black Kites crossing the Himalayas may have physiological adaptations that remain to be investigated.

### Ethics statement

Black Kite trapping and tagging were done in accordance with Art. 44 of the Federal Law No. 52-FZ "On the Animals"—the use of the animals for scientific, cultural, educational, recreational and aesthetic purposes through various forms of observation, tagging, photographing and other research methods without removing the animals from the habitat. In Russian Federation, the Black kite is not classified as protected species and no permits are required for any manipulations with it. Trapping and tagging of birds was performed by trained and experienced person. We performed all methods in accordance with the relevant guidelines and regulations with respect to our study animals. We confirm that the study is reported in accordance with ARRIVE guidelines^76^.

## Data Availability

The datasets used and/or analysed during the current study are available from the corresponding author on reasonable request.

## Abbreviations

KDE: Kernel density estimate
PFA: Post fledging area
TSA: Temporary settlement area
DEM: Digital elevation model
GPS: Global positioning system
SMS: Short message service
GSM: Groupe Spécial Mobile
PCR: Polymerase chain reaction
LMM: Linear mixed model
